# Editorial: Biomaterial advances in intervertebral disc degeneration

**DOI:** 10.3389/fbioe.2023.1153019

**Published:** 2023-02-13

**Authors:** Yong Huang, Ganjun Feng

**Affiliations:** Department of Orthopedic Surgery and Orthopedic Research Institute, West China Hospital, Sichuan University, Chengdu, Sichuan, China

**Keywords:** biomaterial, tissue engineering, drug delivery system, hydrogels, intervertebral disc degeneration

Neck and back pain are ubiquitous in modern society, leading to serious lifelong disability and placing an enormous socioeconomic burden on the global healthcare system. Although the etiology of neck and back pain is multifaceted and still incompletely understood, intervertebral disc degeneration (IDD) is considered to be the most significant contributor. The current treatments for IDD, including medication, surgery, and others, are limited to symptomatic relief but fail to restore the structure and homeostasis of the intervertebral disc. The failure leads to the steady deterioration of compromised discs and undesirable consequences, such as recrudescence or adjacent vertebral disease. Tissue-engineered approaches hold great promise for the treatment of IDD. Gullbrand et al. designed and manufactured a disc-like angle ply structure (DAPS), which has distinct components that mimic the structure of the native disc. The long-term integration and mechanical function of engineered DAPS *in vivo*, even in large animal models have been successfully tested (Gullbrand et al., 2018). After that, Sloan et al. demonstrated that combined nucleus pulposus augmentation using hyaluronic acid injection and annulus fibrosus repair using photo cross-linked collagen patch restore nucleus pulposus hydration, heal annulus fibrosus defects and maintain native torsional and compressive stiffness up to 6 weeks after discectomy injury in a large animal model. These studies move this approach a step towards translational feasibility (Sloan et al., 2020).

Given the indispensable role of biomaterials in tissue engineering for IDD, we prepared this Research Topic to summarize the progress in this field. The harsh microenvironment of IDD is not suitable for disc regeneration. Therefore, various therapeutic agents, including small molecular, growth factor, exosome, and nucleic acids-based drugs are employed to reduce the inflammatory response, promote extracellular matrix synthesis, and direct cell differentiation to create a good regenerative microenvironment. Traditional drug delivery such as systemic administration or *via in situ* injection has low drug availability and high off-target toxicity. Liu et al. have systematically demonstrated that biomaterials-based nano-drug delivery systems have improved treatment results of therapeutic agents for IDD because of their good biodegradability, biocompatibility, precise targeted specific drug delivery, prolonged drug release time, and enhanced drug efficacy (Liu et al., 2023). Except for drug delivery, a good cell carrier is critical for tissue regeneration. The microsphere is a class of three-dimensional spherical structures with an average particle size of 1–1000 μm that could be used in cell carrying and biomedical substance delivery. Guo et al. recently reviewed the use of various microspheres for disc regeneration and clearly demonstrated that the high encapsulation rate and excellent slow-release properties conferred by the porous structure, coupled with good surface adhesion for cell immigration, have led to the gradual emergence of microspheres as the carrier of choice in the field of tissue engineering (Guo et al., 2022). In order to achieve long-term delivery and avoid leakage-associated complications, stimulus-responsive composite hydrogels have been employed. Gao et al. have highlighted the critical role of stimulus-responsive composite hydrogels in disc regenerative medicine with controllable mechanical properties and the ability to achieve liquid-solid phase transition under certain conditions (Gao et al., 2022).

In conclusion, we believe that the studies and reviews on this Research Topic provide new insight into the choice of biomaterials for engineering solutions for intervertebral disc regeneration. We hope to achieve a perfect regenerative repair of the intervertebral disc with the help of emerging material development as soon as possible.
